# Pterostilbene inhibits gallbladder cancer progression by suppressing the PI3K/Akt pathway

**DOI:** 10.1038/s41598-021-83924-4

**Published:** 2021-02-23

**Authors:** Chenhao Tong, Yali Wang, Jiandong Li, Wenda Cen, Weiguang Zhang, Zhiyang Zhu, Jianhua Yu, Baochun Lu

**Affiliations:** 1 Department of Hepatobiliary Surgery, Shaoxing Hospital, Zhejiang University School of Medicine (Shaoxing People’s Hospital), No. 568 Zhongxing North Road, Shaoxing, 312000 Zhejiang China; 2 Shaoxing University School of Medicine, Shaoxing, China; 3 Department of Molecular Medicine and Clinical Laboratory, Shaoxing Second Hospital, Shaoxing, China

**Keywords:** Biliary tract cancer, Biologics, Medicinal chemistry

## Abstract

Gallbladder cancer is the most common malignant tumor of the biliary system and is characterized by difficulty to diagnose in early stages, a high degree of malignancy, and poor prognosis. Finding new drugs may improve the prognosis for this dismal cancer. Herein, we investigated the potential application of pterostilbene (PTS) against gallbladder cancer in vivo and in vitro. PTS potently inhibited cell proliferation, migration and invasion of gallbladder cancer cells. Moreover, PTS also had a function of inducing apoptosis in vitro. Meanwhile, PTS reversed EMT with a correlated inhibition of PI3K/Akt activation. Tumor xenograft models showed that PTS inhibited tumor growth and had low toxicity in vivo*,* which were consistent with the in vitro data. These findings indicate that PTS arrests cell growth through inhibition of PI3K/AKT signaling and is a potential drug for the therapy of gallbladder cancer.

## Introduction

Gallbladder cancer (GBC) is a malignant tumor derived from epithelial cells of the gallbladder and has a poor prognosis^1^. While GBC is relatively rare in Western countries, it is more common in developing countries such as East Asia, India, and South America. A recent study showed that GBC accounted for 0.76–1.2% of all cancers in China^2^. Over the same period, GBC accounted for 0.4–3.8% of all bile duct diseases in China, ranking the 6th most common among digestive tract tumors. The 5-year overall survival rate of GBC patients is only 5%^3,4^. In 2014, the incidence of GBC was 3.82/100,000, the bid-winning incidence was 2.38/100,000, and the cumulative incidence was 0.27% in China^5^. Most high recurrence rate following potentially curative surgery^6,7^. The lack of effective drugs is another important factor that contributes to the dismal prognosis of GBC. Although gemcitabine-based chemotherapy has been reported to improve the prognosis of GBC patients^8–10^, its actual therapeutic effect in the clinic is far from satisfactory^11^. Thus, finding new potential drugs and identifying their underlying mechanism of activity could improve outcomes for this aggressive cancer type.

Pterostilbene (trans-3,5-dimethoxy-4′-hydroxystilbene; PTS) is extracted from blueberries, grapes, and palmettos^12,13^. PTS can act on a variety of signaling pathways to inhibit tumor cell proliferation^12,14,15^. Previous studies have shown that PTS scavenges reactive oxygen species and blocks ultraviolet-induced skin cancer^16^. Different PTS concentrations show different effects on cancer cells^17–19^. High concentration PTS can cause a G1 cell cycle arrest, while low concentration PTS can induce a cell cycle block at S phase, although the mechanism is unclear^15^. Additionally, PTS can induce apoptosis in various tumor cells, including cancers of the bladder^20^, lung^17^, breast^12,13^, prostate^14^, lymphomas^21^, and oral mucosa^22^.

Epithelial-mesenchymal transition (EMT) is necessary for many physiological processes but can also lead to pathological fibrosis and cancer progression^23–25^. Previous studies have shown that EMT is an important mechanism that promotes the growth and stemness of GBC cells^26^. Generally, elevated levels of mesenchymal markers and decreased expression of epithelial markers are found in cells that have undergone EMT.

The PI3K/Akt signaling pathway is an important regulatory pathway in cells that controls survival, metabolism, growth, differentiation, and skeletal recombination^27^. Multiple growth factors and oncogenes affect the PI3K/Akt pathway, including the insulin receptor tyrosine kinase, the epidermal growth factor receptor, the related insulin-like growth factor 1 receptor, and platelet-derived growth factor receptors^28^. Dysregulation of the PI3K/Akt pathway leads to abnormal changes in multiple downstream effectors, resulting in abnormal cell proliferation, differentiation, and metabolism^28^.

We hypothesized that PTS could be a potential treatment for GBC. The activities of PTS were evaluated by studying the characteristics of GBC cells with and without PTS treatment, including their proliferation, migration, and survival. Furthermore, the PI3K/Akt pathway was identified as the underlying mechanism for the function of PTS in GBC cells.

## Results

### PTS inhibited the proliferation of GBC cells

**Figure 1:**
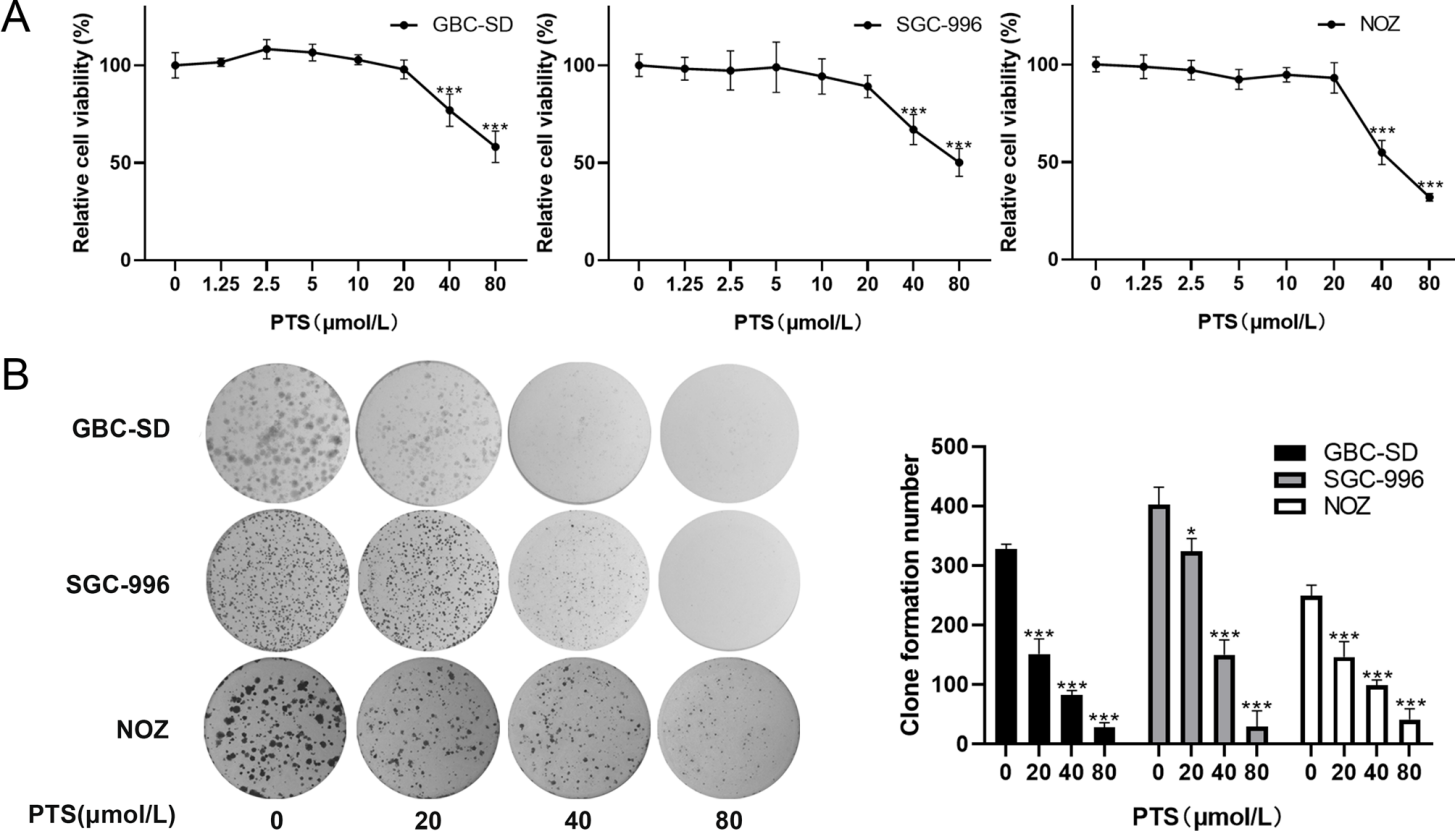
PTS decreased the viability of human GBC cells. (**A**) The CCK8 assay showed that PTS induced a dose-dependent cytotoxicity in GBC-SD, SGC-996 and NOZ cells. (**B**) Colony formation assays showed that the colony formation ability of GBC-SD, SGC-996 and NOZ cells were decreased with increasing PTS concentrations. *P < 0.05; **P < 0.01; ***P < 0.001, compared with the control group.

The effect of PTS on the proliferation of GBC-SD, SGC-996 and NOZ cells was determined by CCK8 assays. The results showed that PTS had a dose-dependent inhibitory effect on the proliferation of GBC-SD, SGC-996 and NOZ cells (*P* < 0.001, Fig. 1A). The sensitivity of the three cell lines to PTS were similar (Fig. 1A). Colony formation assays also showed that PTS inhibited colony formation in both GBC-SD, SGC-996 and NOZ cells compared with control groups (Fig. 1B). Proliferating cell nuclear antigen (PCNA) is a marker of cell proliferation^29^, and western blot analysis showed that GBC-SD, SGC-996 and NOZ cells had lower PCNA levels following PTS treatment (Fig. 4C). These results suggested that PTS inhibited the proliferation of GBC cells.

### PTS induced apoptosis in GBC cells

**Figure 2:**
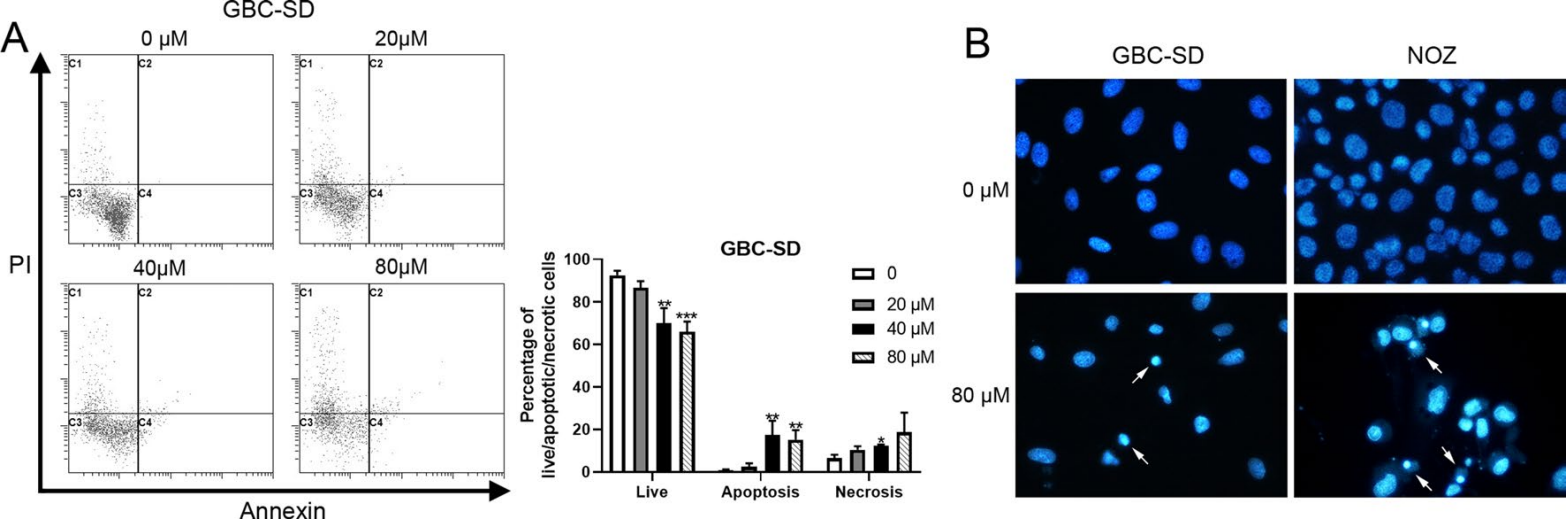
PTS increased apoptosis in human GBC cells. (**A**) The effect of PTS on the percentages of alive, apoptotic, and necrotic GBC-SD cells was evaluated by Annexin V/PI apoptosis assays. The mean values of the percentage of alive, apoptotic, and necrotic cells from independent experiments ± SD are presented. (**B**) Hoechst staining of GBC-SD and NOZ cells treated with PTS for 48 h. White arrows highlight apoptotic cells. *P < 0.05; **P < 0.01; ***P < 0.001, compared with the control group.

Anticancer drugs display antineoplastic activity by inhibiting proliferation and inducing apoptosis. Thus, we next examined whether PTS induced apoptosis in GBC cells. Apoptosis was detected using the Annexin V/PI apoptosis assay. The results showed that the number of apoptotic cells increased significantly while the number of live cells decreased when GBC-SD cells were treated with 40 or 80 μM PTS (*P* < 0.01, Fig. 2A). Hoechst 33,342 staining showed that the nuclei of PTS-treated GBC-SD and NOZ cells were concentrated and bright, while cells in the control group showed no apoptotic nuclei (Fig. 2B). These results indicated that PTS promoted apoptosis in GBC cells.

### PTS inhibited the migration and invasion of GBC cells

**Figure 3:**
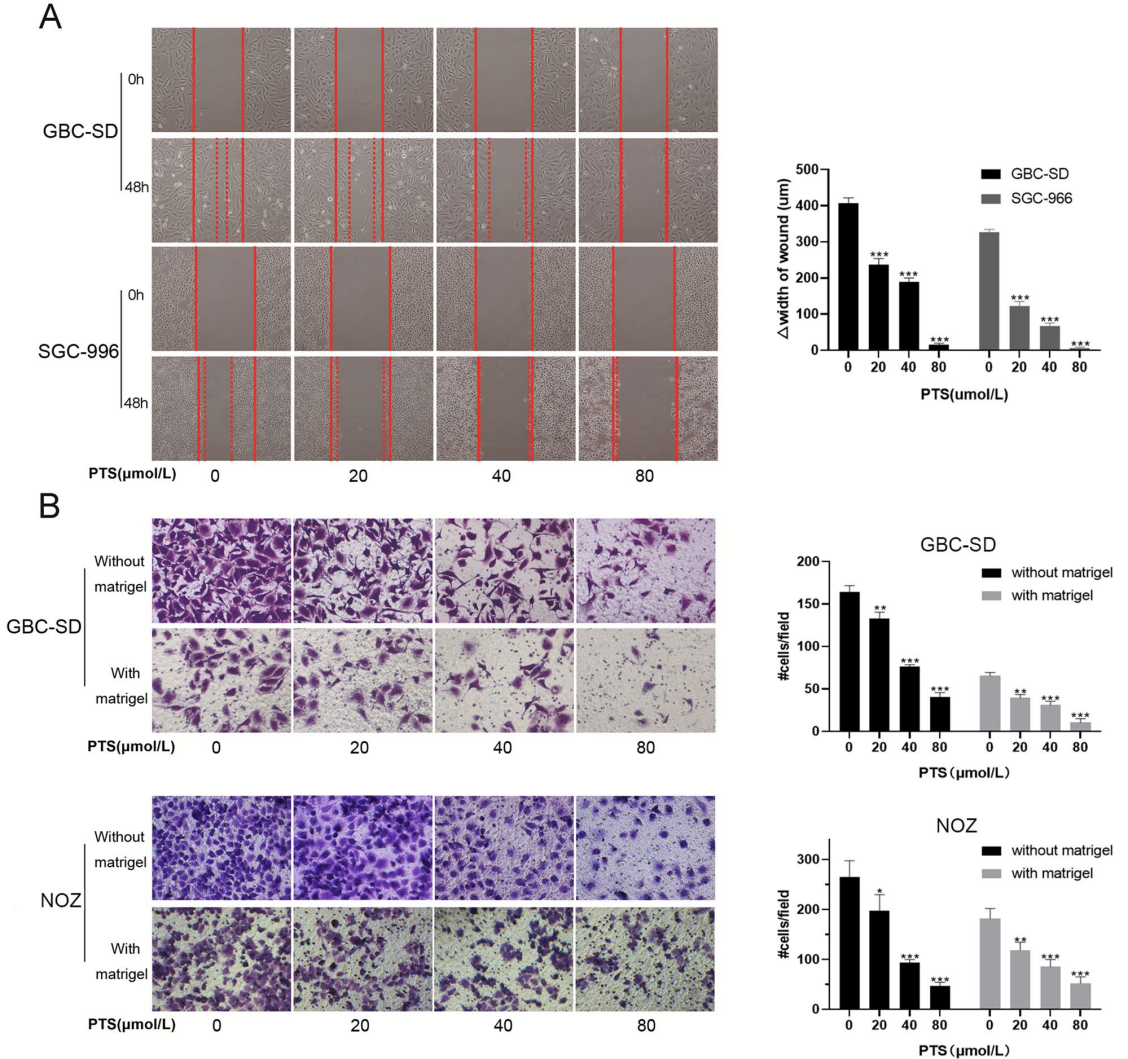
PTS treatment reduced the migration and invasion capabilities of human GBC cells. (**A**) Scratch assays showed that the migration ability of GBC-SD and SGC-996 cells were inhibited by 48-h treatment with different concentrations of PTS. (**B**) The effects of PTS on the migration and invasion of GBC-SD and NOZ cells in vitro. Cells were counted in three random fields at 400 × magnification after a 48-h incubation. *P < 0.05; **P < 0.01; ***P < 0.001, compared with the control group.

Scratch assays were performed to investigate the effect of PTS on the migration of GBC cells. With increasing PTS concentrations, the distance between the scratch edges after 48-h incubation gradually increased in GBC-SD and SGC-996 cells. (*P* < 0.001, Fig. 3A). Moreover, the Transwell assay results showed that the migration and invasion capabilities of GBC-SD and NOZ cells were significantly inhibited with different PTS concentrations compared with the control group (Fig. 3B). Moreover, a concentration dependence was observed after PTS treatment. Together, these data indicated that PTS inhibited the migration and invasion of GBC cells.

### PTS reversed EMT by inhibiting PI3K/Akt signaling in GBC cells

**Figure 4:**
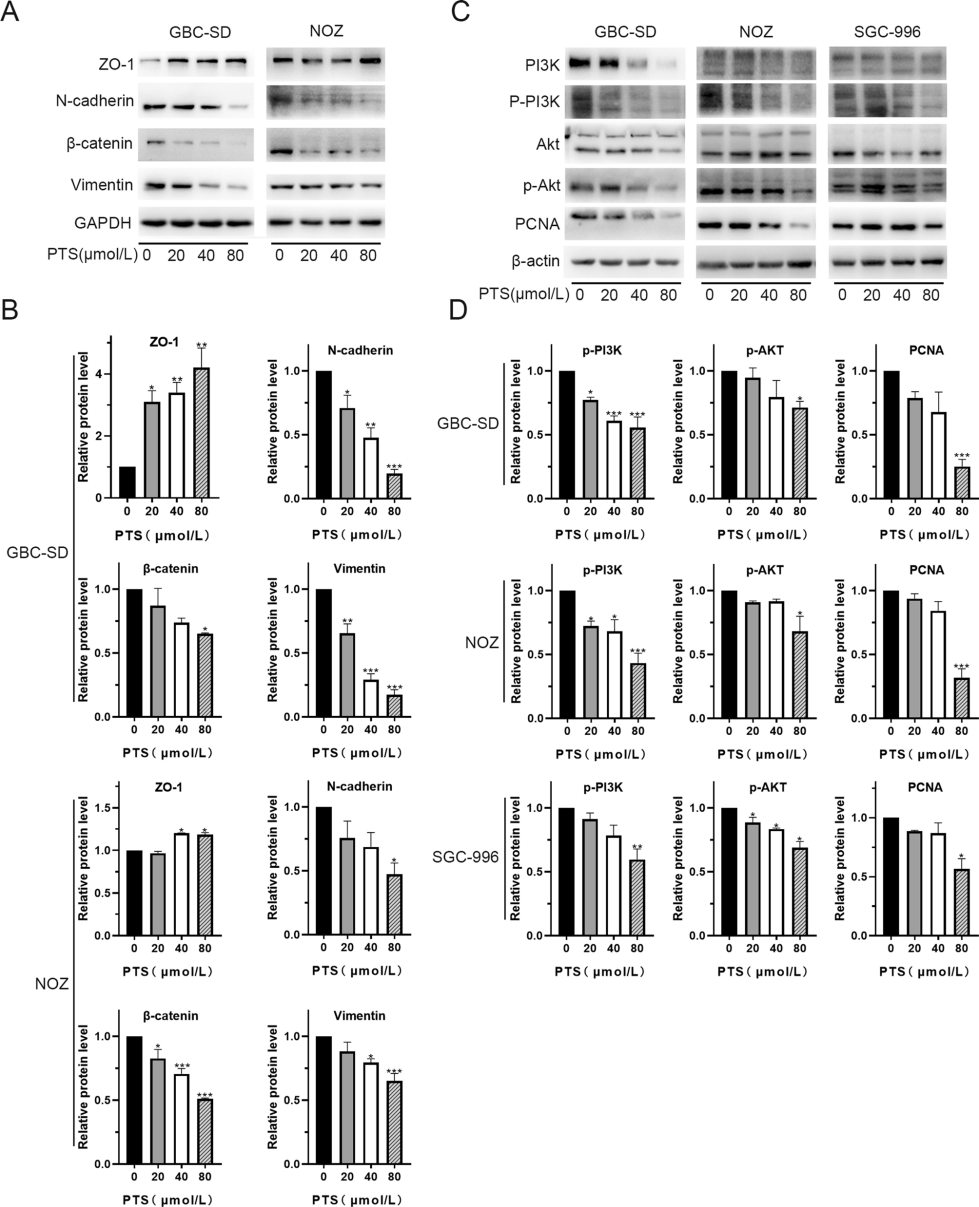
PTS treatment regulates signaling through the PI3K/Akt pathway. (**A**) N-cadherin, Vimentin, and β-catenin were decreased, while ZO-1 expression was significantly increased in PTS-treated GBC-SD and NOZ cells. (**B**) Quantification of EMT related-protein levels in blot. (**C**) Western blot analysis showed that the levels of PCNA, Akt, p-Akt, PI3K, p-PI3K were down-regulated in PTS-treated GBC-SD, SGC-996 and NOZ cells. (**D**) Quantification of PI3K/Akt related-protein levels in blot. *P < 0.05; **P < 0.01; ***P < 0.001, compared with the control group.

Cancer cells show enhanced migration and invasion behaviors following EMT. Thus, we next investigated the levels of EMT-associated proteins by western blot in GBC-SD cells following PTS treatment. Mesenchymal-associated proteins, including N-cadherin, Vimentin and β-catenin were downregulated in GBC-SD and NOZ cells after 48-h PTS treatment, whereas the epithelial adhesion molecule ZO-1 was upregulated (Fig. 4A,B).

The PI3K/Akt regulates EMT in various cancers^30,31^. Our results showed that the key proteins in the PI3k/Akt pathway, including PI3K, p-PI3K, AKT and p-Akt. p-PI3K and p-Akt were both significantly downregulated in GBC-SD, SGC-966 and NOZ cells following PTS treatment (Fig. 4C,D).

### PTS inhibited the formation of GBC xenografts in vivo

**Figure 5:**
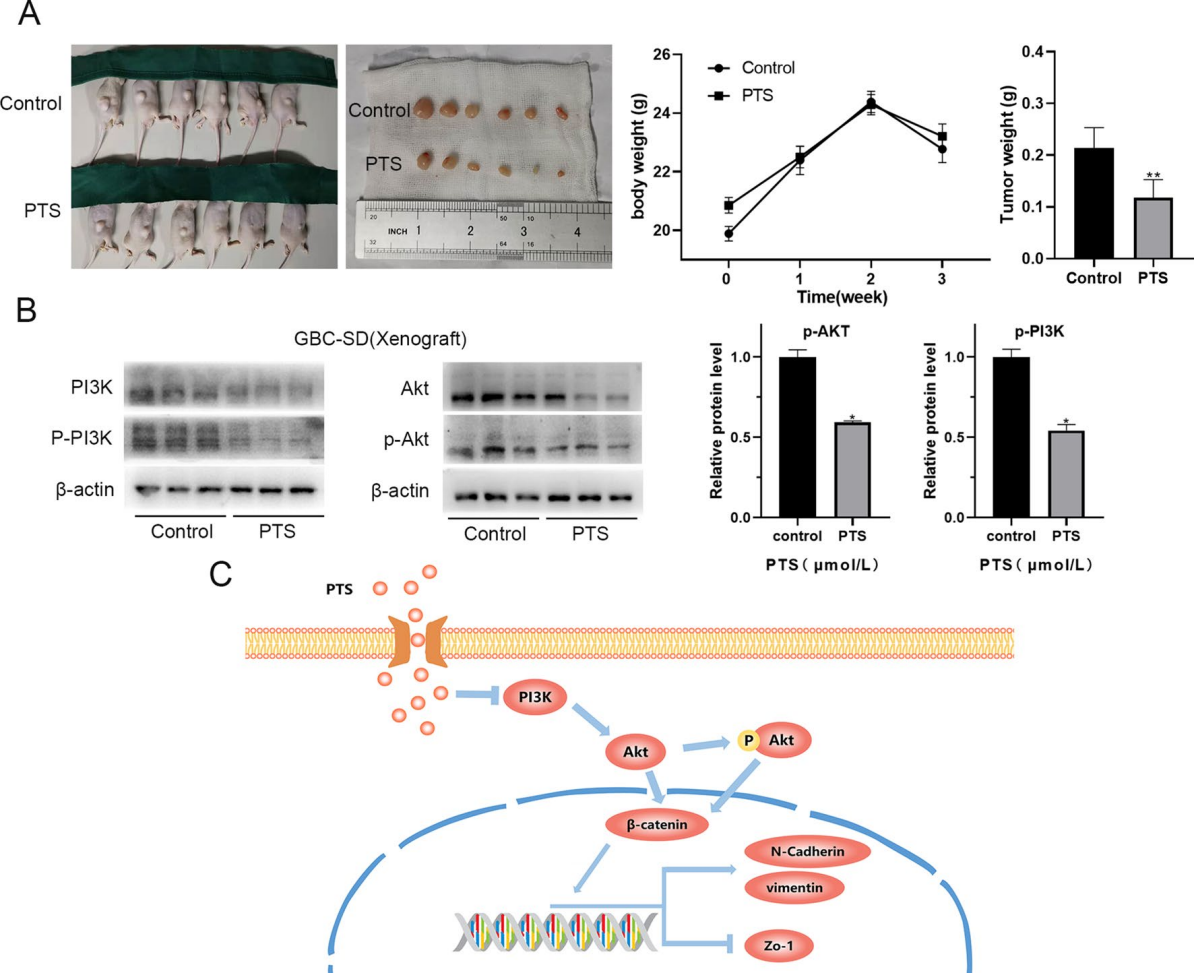
(**A**) The anti-tumor effect of PTS in the GBC-SD xenograft model. Body weights of GBC-SD xenograft-bearing mice after PTS treatment. Tumor weights and size of GBC-SD xenografts treated with PTS. (**B**) Expression of PI3K, p-PI3K, Akt and p-Akt protein in GBC-SD xenografts. (**C**) A proposed model for how PI3K/Akt/β-catenin signaling mediates EMT in GBC-SD cells. *P < 0.05; **P < 0.01, compared with the control group.

To further evaluate the potential application of PTS in vivo, xenograft formation in nude mice was investigated. The results showed that intraperitoneal injection of PTS significantly inhibited tumor growth in vivo*,* whereas PTS did not have a remarkable influence on the body weight of tumor-bearing mice (Fig. 5A).

Then, the xenograft tumors were excised and PI3K/Akt pathway related proteins levels were examined by western blot. We found that p-PI3K and p-Akt were also significantly down-regulated in tumor tissues (*P* < 0.05, Fig. 5B). It indicated that inhibiting PI3k/Akt pathway was the potential mechanisms of how PTS regulated the plasticity of GBC cells (Fig. 5C).

## Discussion

Over the past 20 years, advancements in the efficacy of anticancer treatments have improved the prognosis of some cancers, including breast and colon cancer^32–34^. However, GBC does not belong this series and still has an unimproved prognosis. Thus, by exploring new potential drugs and evaluating their activity in preclinical models, we hope to improve the prognosis of GBC.

Some plant extracts have been used in the clinic to treat cancer, including paclitaxel and hydroxycamptothecin^35,36^. Resveratrol (3,4′,5-trihydroxydistyrene) is found in blueberries, grapes, palmettos, and other plants^37,38^. PTS is a dimethyl derivative of resveratrol (3,5-dimethoxyl-4′-hydroxystyrene) that has similar biological activities^39^. Its function including anti-dyslipidemia, cardiovascular protection and neuroprotective effects^12,13^. As an extracted monomer of plants, PTS has also been proven to have antitumor effects in various cancers, including cancers of the bladder^20^, lung^17^, breast^12,13^, prostate^14^, lymphomas^21^, and oral mucosa^22^. The potential mechanisms of PTS include inhibiting tumor cell proliferation and migration^15,20,21^, inducing cell cycle arrest^12,21^, accelerating apoptosis^12,17,21,22^, and inhibiting metastasis^40,41^. Moreover, PTS can inhibit the EMT process of breast cancer with the long non-coding RNAs^42^. In this study, we investigated the effects of PTS on GBC in vivo and in vitro.

Uncontrolled growth and suppression of apoptosis help cancer cells achieve rapid growth^43^. Anticancer drugs usually work by inhibiting cell proliferation and promoting apoptosis. Our results showed that PTS inhibited proliferation and accelerated apoptosis in GBC cells. Various signaling pathways have been invoked to explain the mechanism of the antitumor function of PTS in different cancers^14,15,44^, including Akt-related pathways. It has been confirmed that PTS inhibits the proliferation of lung cancer cells in mice by inhibiting epidermal growth factor signaling, which reduces downstream Akt phosphorylation^14,45^. Other studies showed that PTS inhibited obesity-related colorectal cancer and attenuated mantle cell lymphoma progression by regulating PI3K^46,47^. The PI3K/Akt pathway is known to play important roles in cancer cells^48^. The PI3Ks are a family of lipid kinases that transfer signaling proteins to cell membranes by producing phospholipids. These signaling proteins include Akt, which is recruited to cell membranes and phosphorylated at SER473^49^. Once phosphorylated, Akt regulates various biological responses through diverse downstream molecules^50^. Previous studies have shown that PTS inhibits the expression of epidermal growth factor receptor, thereby reducing p-Akt levels, which leads to decreased activation of the G- and S-phases of the cell cycle, ultimately inhibiting the proliferation of lung cancer cells. Similarly, in our study, PI3K and p-Akt protein levels were significantly decreased in GBC cells following PTS treatment. These results suggest that PTS inhibits proliferation and induces apoptosis in GBC cells by decreasing PI3K/Akt signaling.

Several studies have confirmed that PI3K signaling is closely related to EMT^31,51^. EMT is the process through which epithelial cells acquire the ability to migrate; EMT is also associated with cellular dedifferentiation. Through EMT, cells lose their polarity as well as connections to basement membranes. Following EMT, tumor cells acquire the ability to resist apoptosis and degrade the extracellular matrix, and thus display mesenchymal phenotypes, such as increased migration and invasion^52^. EMT of GBC cells is regulated through the PI3K/Akt pathway^53,54^. Previous studies have shown that PTS inhibits PI3K and Akt, and increases β-catenin phosphorylation, promoting its degradation^55^. Taken together, Akt phosphorylation activates a series of changes that accelerate EMT. It is worth noting that in our study, PTS inhibited the metastasis of GBC cells and regulated the expression of EMT makers by decreasing PI3K-Akt signaling activity. While PTS significantly increased the expression of ZO-1, treatment decreased the expression of Vimentin and N-cadherin. ZO-1 is necessary for tight junction formation and function, is involved in regulating paracellular permeability and cell polarity, and preventing transmembrane proteins from moving between the apical and basolateral cell surfaces^56^. Vimentin is a predictive biomarker for tumor growth and metastasis^57^ that is closely related to the invasion and metastasis of various malignant tumors^58^. As one of the important EMT markers, N-cadherin plays a key role in the invasion and metastasis of many tumors. These changes are considered a common phenomenon in EMT, and N-cadherin expression can be used as a biological indicator to judge prognosis^59,60^. The above proteins play an important role in the evolution of EMT, and the characteristic changes in these proteins were verified in our study. Together, these data indicate that PTS regulates the plasticity of GBC cells via reducing PI3K/Akt signaling.

In conclusion, PTS inhibited the proliferation, apoptosis, migration, and invasion of human GBC cells, which may be related to inhibition of the PI3K/Akt pathway.

## Materials and methods

### Chemicals and reagents

PTS (C_16_H_16_O_3_; > 98% pure) was purchased from Shanghai Yuanye Biotechnology Company (Cat No. B21702, Shanghai, China). Cell Counting Kit-8 (CCK-8) was obtained from MCE (Monmouth Junction, NJ, USA). Annexin V Apoptosis Detection Kit was purchased from BD Biosciences (Franklin Lakes, NJ, USA). Primary antibodies against phospho-Akt (Ser473), p-PI3 Kinase, PI3 Kinase, ZO-1, and N-Cadherin were purchased from Cell Signaling Technology (Danvers, MA, USA). Primary antibodies against Akt, and GAPDH were purchased from Proteintech (Wuhan, China).

### Cell culture

The human GBC cell line GBC-SD was obtained from the Cell Bank of Type Culture Collection of the Chinese Academy of Sciences (Shanghai, China); SGC-996 cells were obtained from Dr. Ying-Bin Liu’s lab at Xin Hua Hospital Affiliated to Shanghai Jiao Tong University School of Medicine (China). NOZ cells were obtained from the HAKATA Cell Bank of the Shanghai Chuanqiu Biotechnology Co., LTD (Shanghai, China). GBC-SD and SGC-996 cell lines were cultured in RPMI‑1640 and NOZ cell lines were cultured in DMEM medium with 10% fetal bovine serum (both from Gibco, Waltham, MA, USA) in a humidified atmosphere containing 5% CO_2_ at 37 °C. PTS was dissolved in DMSO and diluted in RPMI-1640 or DMEM (with same level of DMSO in controls) to a final value of DMSO < 0.1%.

### Cell viability assays

The proliferation of GBC-SD, SGC-996 and NOZ cells under PTS treatment was evaluated by the CCK-8 assay. Briefly, cells in logarithmic growth phase were inoculated in 96-well plates with 5 × 10^3^ cells per well and cultured overnight. The cells were treated with different concentrations of PTS (0–80 µmol/L) for 48 h, and five parallel wells were used for each group. After treatment, 10 µL of CCK8 solution were added to each well and incubated at 37 °C for 1 h. The absorbance value of each well was detected at 450 nm with an enzyme label instrument (Molecular Devices Co., San Jose, CA, USA), and cell viability was analyzed using GraphPad Prism 5.0 (GraphPad Software Inc., La Jolla, CA, USA).

### Scratch assays

The migration of GBC-SD and SGC-996 cells were examined by scratch assays. Briefly, cells were seeded and grown to confluence on 35-mm cell culture dishes. Scratches were then made with a sterile 200-µL pipet tip, and the plates were washed three times with PBS. Then serum-free medium contained different concentrations of PTS was added. Images were captured at 0, 24, and 48 h to record the scratch widths. Images were obtained with a Nikon light microscope system (Tokyo, Japan).

### Colony formation assays

GBC-SD, SGC-996 and NOZ cells were individually seeded in 6-well plates at 5 × 10^2^ cells per well and cultured overnight. The cells were treated with different concentrations of PTS (0–80 µmol/L) for 10 days, with the medium replaced every 2 days. After 10 days, the supernatant was discarded, the cells were fixed with 4% paraformaldehyde and stained with 0.1% crystal violet, and then colonies of more than 50 cells were counted using a Nikon light microscope.

### Invasion and migration assays

Chambers with (*invasion assays*) or without (*migration assays*) Matrigel coating that fit into 24-well plates were purchased from Corning Inc. (Corning, NY, USA). The GBC cells were washed twice with PBS, and then resuspended in serum-free medium at a concentration of 2 × 10^5^ cells/mL in the presence or absence of PTS. Next, 100 µL of the cell mixtures were added to the upper well of the invasion chambers. Serum-containing medium (600 µL) was added to the lower chamber of each well. After incubation for 48 h, cotton swabs were used to wipe off cells in the upper chamber, and 0.1% crystal violet was used to stain the remaining cells after formaldehyde fixation. Mean values of the number of migrated cells in 10 random fields under the microscope were calculated, as well as the standard deviation.

### Apoptosis analysis

Apoptosis was analyzed by two methods: flow cytometry and Hoechst 33,342 staining. For flow cytometry experiments, GBC-SD cells were incubated with different concentrations of PTS for 48 h. After washing twice with cold PBS, the cells were resuspended in binding buffer at a concentration of 1 × 10^6^ cells/mL, and then mixed with 5 μL of propidium iodide (PI) and 5 μL FITC Annexin V. After incubating for 30 min at room temperature in the dark, the cells were analyzed by a FACScan (Beckman Coulter Inc., Brea, CA, USA). For Hoechst 33,342 staining, GBC-SD and NOZ cells were inoculated in 6-well plates and treated with different concentrations of PTS for 2 days. The cells were stained with Hoechst 33,342 (Beyotime Institute of Biotechnology, Nanjing, China) and observed under a Nikon fluorescence microscope.

### Western blot analysis

GBC-SD, SGC-996 and NOZ cells were incubated for 48 h with different concentrations of PTS, and then total protein was extracted with lysate buffer (Beyotime) containing Phenylmethanesulfonyl fluoride (Beyotime). GBC-SD xenografts were ground in liquid nitrogen. Cytoplasmic proteins were harvested by sonication and centrifugation. The BCA kit (Beyotime) was used to determine protein concentrations. 20 μg total protein was separated on 10–12% SDS‑PAGE gel and was transferred onto a polyvinylidene fluoride membranes (Merck KGaA, Temecula, CA, USA). Membranes were blocked in 5% skim milk for 1 h at 25 °C and incubated with primary antibodies at 4 °C overnight. Membranes were washed three times with TBST and incubated with the horseradish peroxidase-conjugated secondary antibodies (1:2000; Beyotime) at room temperature for 1 h. Immunoreactive bands were detected with the ECL kit (Millipore, Temecula, CA, USA) and quantified by Quantity One (Bio‑Rad, Hercules, CA, USA).

### Animal experiments

Male athymic nude mice (6-weeks-old) were purchased from SLAC Laboratory Animal Company (Shanghai, China). All procedures were approved by the Animal Care and Use Committee of Shaoxing People’s Hospital and conformed to the ARRIVE guidelines 2.0 published in PLOS Biology. The nude mice had ad libitum access to food and water and were maintained at 20˚C, with 50% humidity under 12-h light/dark cycles. The buttocks of all nude mice were subcutaneously injected with 100 μL of PBS containing GBC-SD cells (1 × 10^6^). Tumor xenografts appeared approximately 4–6 d following cell injection. Mice were randomly divided into two groups (N = 6). The control group was intraperitoneally injected with vehicle solution (0.9% saline, 20% DMSO) while the experimental group was injected with PTS (30 mg/kg, dissolve in 20% DMSO). PTS treatments were initiated at 7d after cell injection and performed once every 2 days for 3 weeks. The weights of the mice were measured weekly, and the mice were sacrificed after 3 weeks to isolate tumors and record their weights.

### Statistical analysis

All experiments were repeated at least three times, and data are presented as means ± SD. Student’s t-test was used to determine statistical significance between two groups. One-way ANOVA followed by the Tukey–Kramer adjustment was used to examine differences among multiple groups. All statistical analyses were conducted using SPSS v20.0 (IBM, Armonk, NY, USA) and *P* < 0.05 was considered statistically significant.

## Supplementary Information

Supplementary Information.
