# Supplementary material for: Pterostilbene inhibits gallbladder cancer progression by suppressing the PI3K/Akt pathway
Source: Sci Rep. 2021 Feb 23;11:4391. doi: 10.1038/s41598-021-83924-4 (PMC7902850; doi:10.1038/s41598-021-83924-4)
Supplement: Supplementary file 1 — Supplementary Information. [file 41598_2021_83924_MOESM1_ESM.pdf]

Pterostilbene inhibits gallbladder cancer progression by suppressing the PI3K/Akt pathway

Chenhao Tong[1*], Yali Wang[1*], Jiandong Li[1], Wenda Cen[2], Weiguang Zhang[3], Zhiyang Zhu[1], Jianhua Yu[1,2✉], Baochun Lu[1,2✉].

[1]Department of Hepatobiliary Surgery, Shaoxing Hospital, Zhejiang University School of Medicine (Shaoxing People's Hospital), Shaoxing, China.

[2]Shaoxing University School of Medicine, Shaoxing, China.

[3]Department of Molecular Medicine and Clinical Laboratory, Shaoxing Second Hospital, Shaoxing, China.

*These authors contributed equally to this work.

✉Corresponding author.

Correspondence to: Dr. Jianhua Yu & Dr. Baochun Lu, Department of Hepatobiliary Surgery, Shaoxing Hospital, Zhejiang University School of Medicine (Shaoxing People's Hospital), No. 568 Zhongxing North Road, Shaoxing, Zhejiang 312000, China.

E-mail: yujianhua@zju.edu.cn (Dr. Jianhua Yu); sygd_lbc@126.com (Dr. Baochun Lu).

Supplementary information

Figure 1

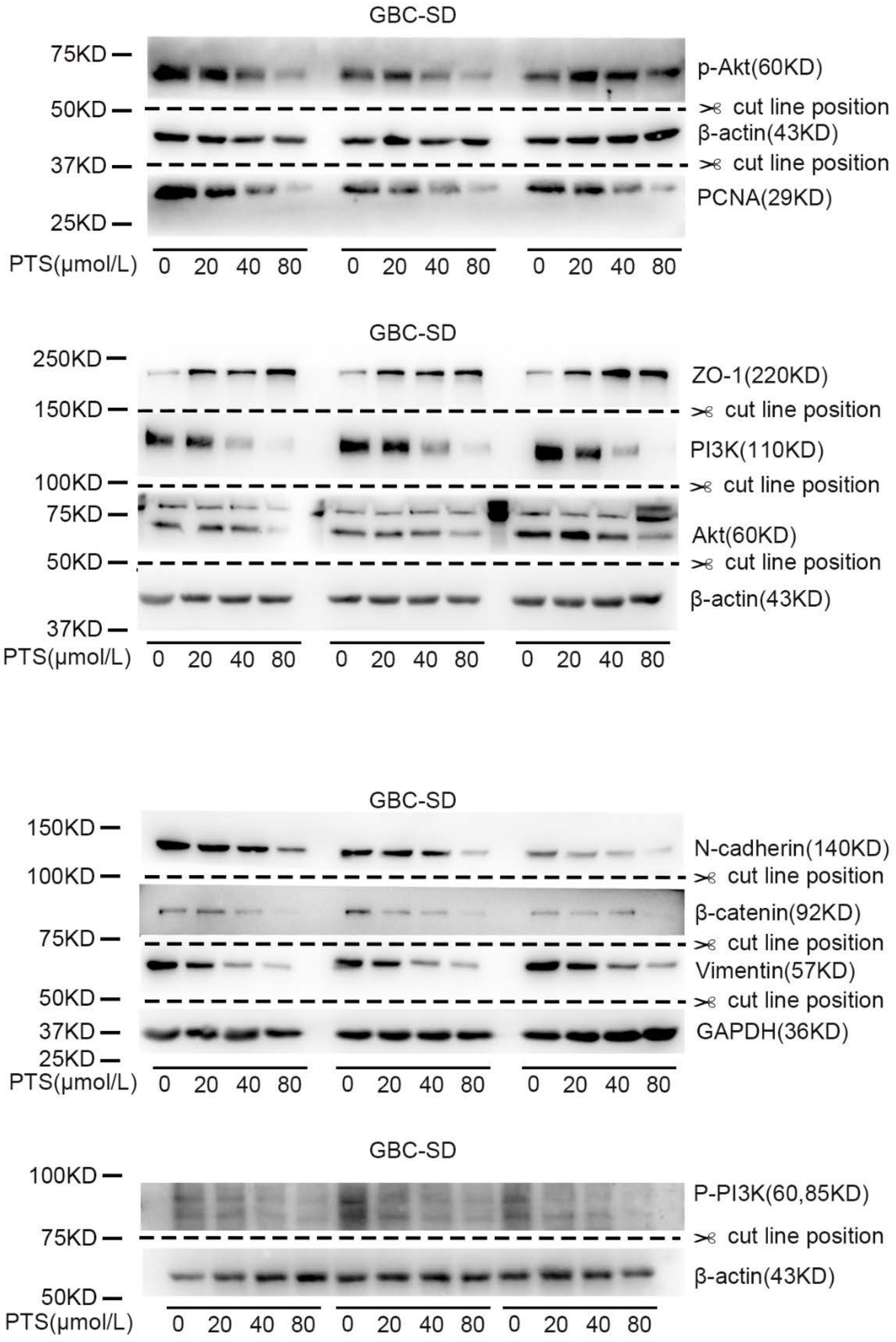

Fig.1. PTS treatment regulates signaling through the PI3K/Akt pathway. Western blot analysis showed that the levels of PCNA, Akt, p-Akt, PI3K, p-PI3K, N-cadherin, Vimentin and β-catenin were decreased, while ZO-1 expression was significantly increased in PTS-treated GBC-SD cells.

Figure 2

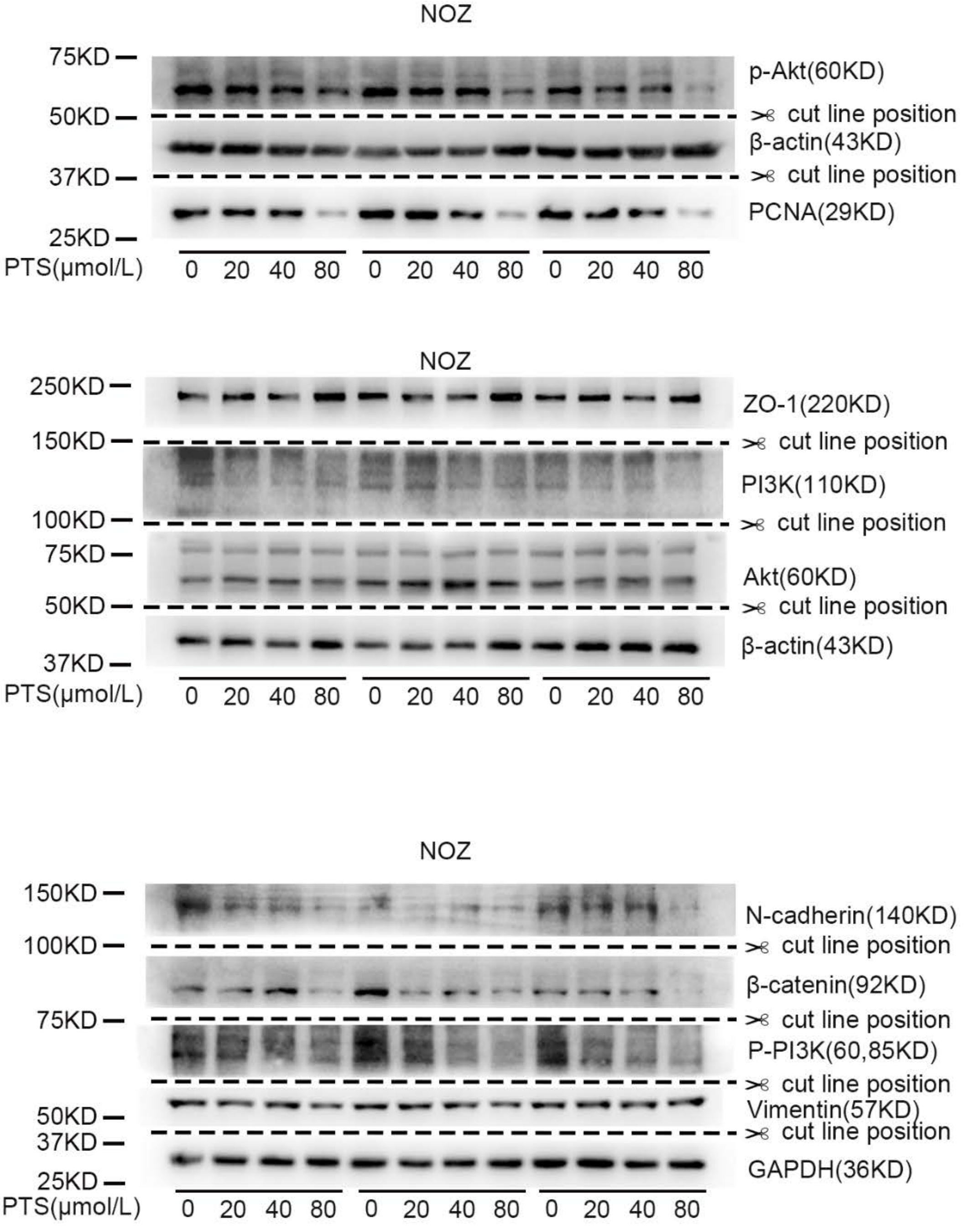

Fig.2. PTS treatment regulates signaling through the PI3K/Akt pathway. Western blot analysis showed that the levels of PCNA, Akt, p-Akt, PI3K, p-PI3K, N-cadherin, Vimentin and β-catenin were decreased, while ZO-1 expression was significantly increased in PTS-treated NOZ cells.

Figure 3

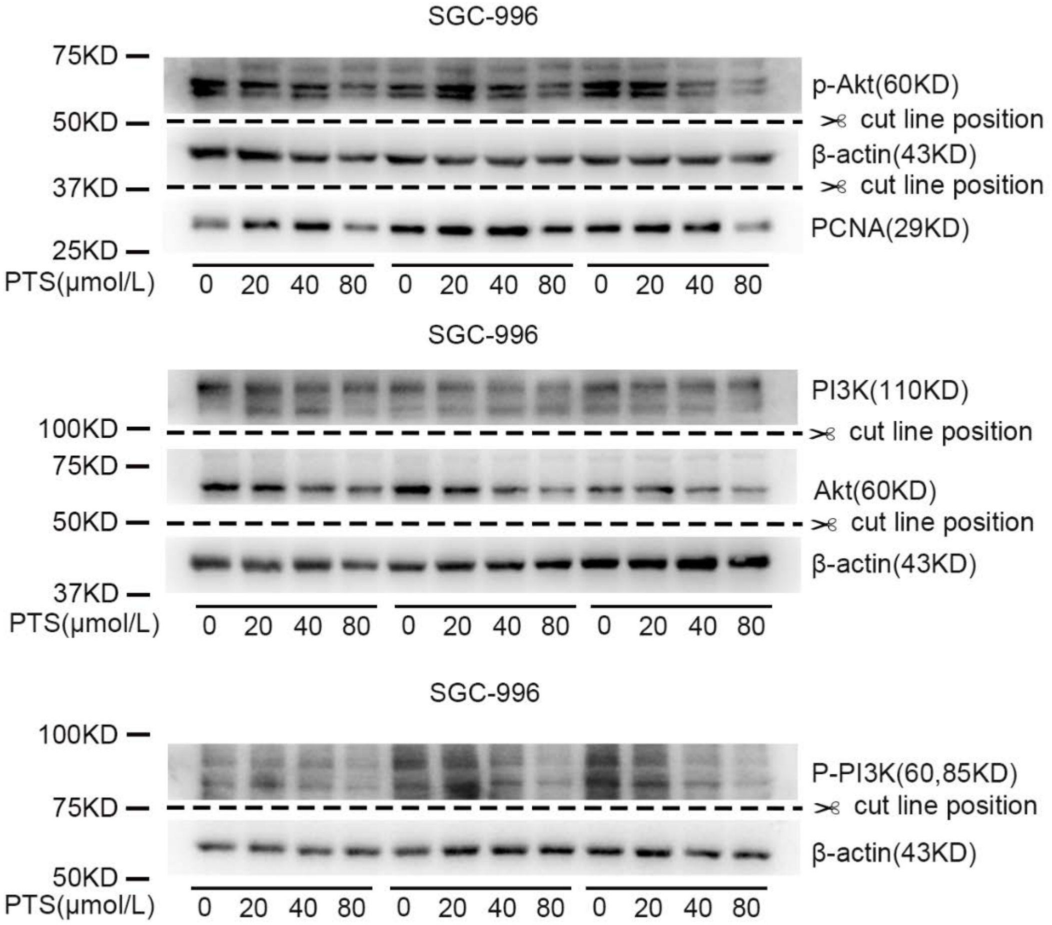

Fig.3. PTS treatment regulates signaling through the PI3K/Akt pathway. Western blot analysis showed that the levels of PCNA, Akt, p-Akt, PI3K and p-PI3K were decreased in PTS-treated SGC-996 cells.

Figure 4

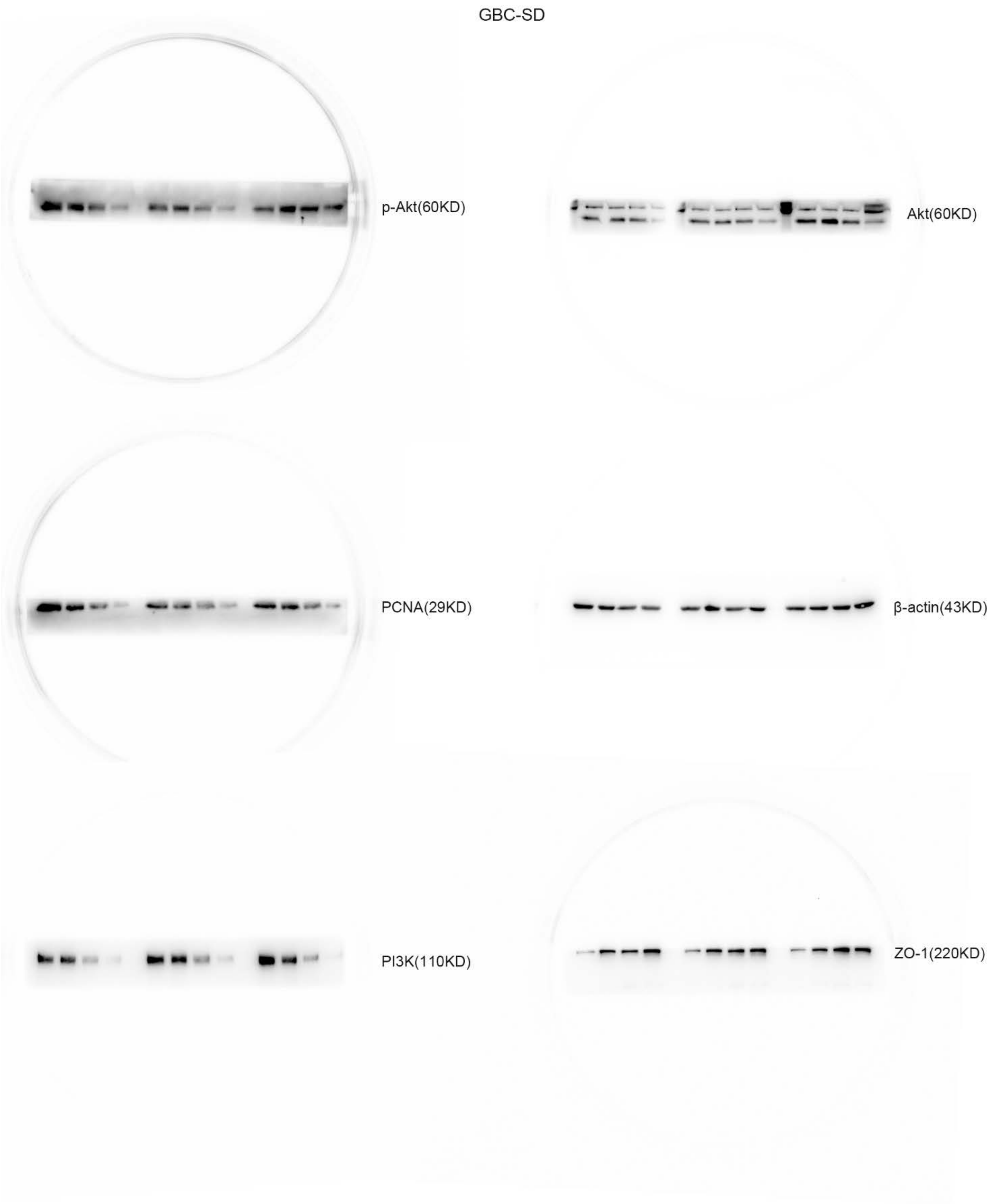
GBC-SD
p-Akt(60KD)
Akt(60KD)
PCNA(29KD)
β-actin(43KD)
PI3K(110KD)
ZO-1(220KD)

Figure 5

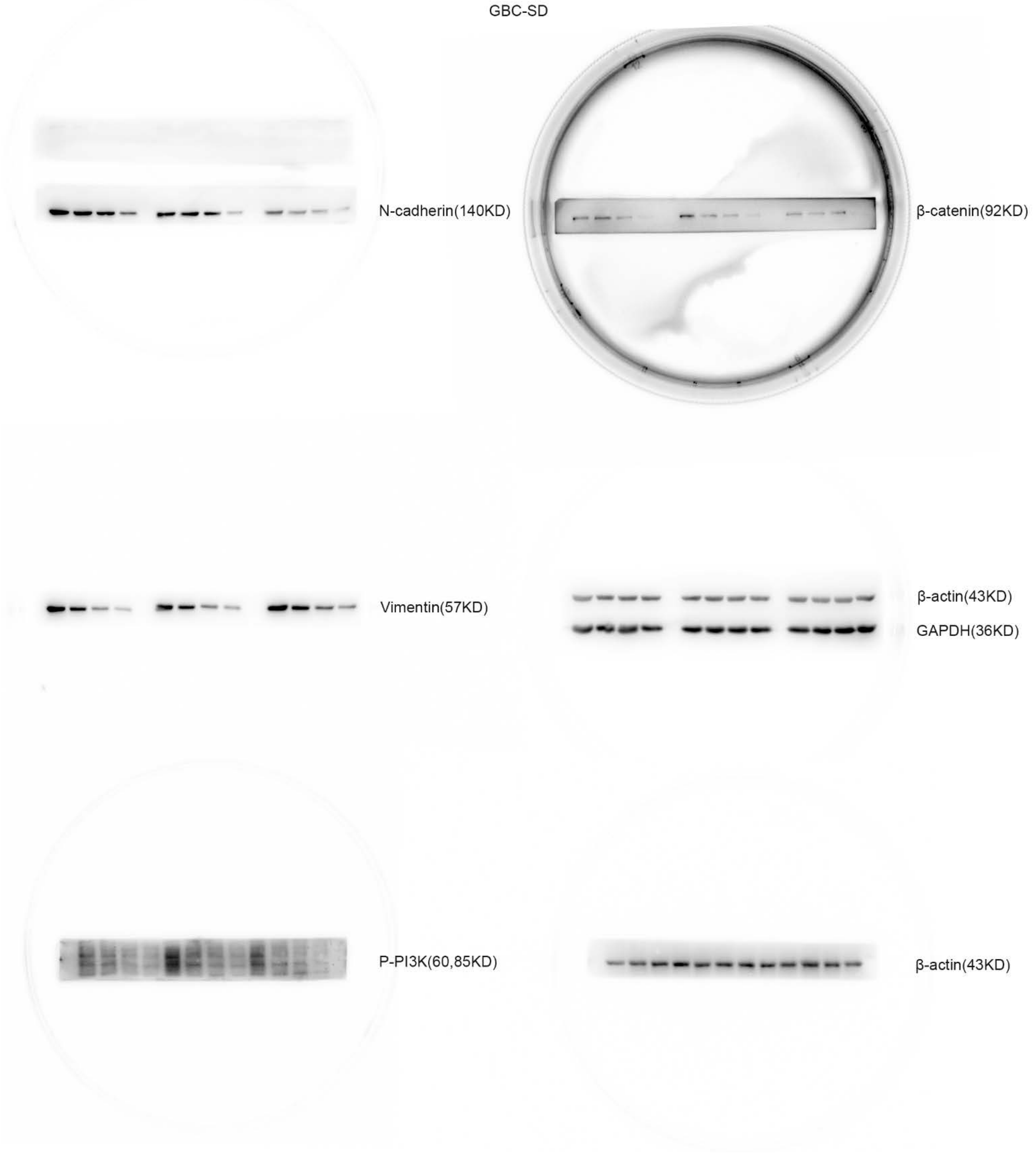
GBC-SD
N-cadherin(140KD)
β-catenin(92KD)
Vimentin(57KD)
β-actin(43KD)
GAPDH(36KD)
P-PI3K(60,85KD)
β-actin(43KD)

Figure 6

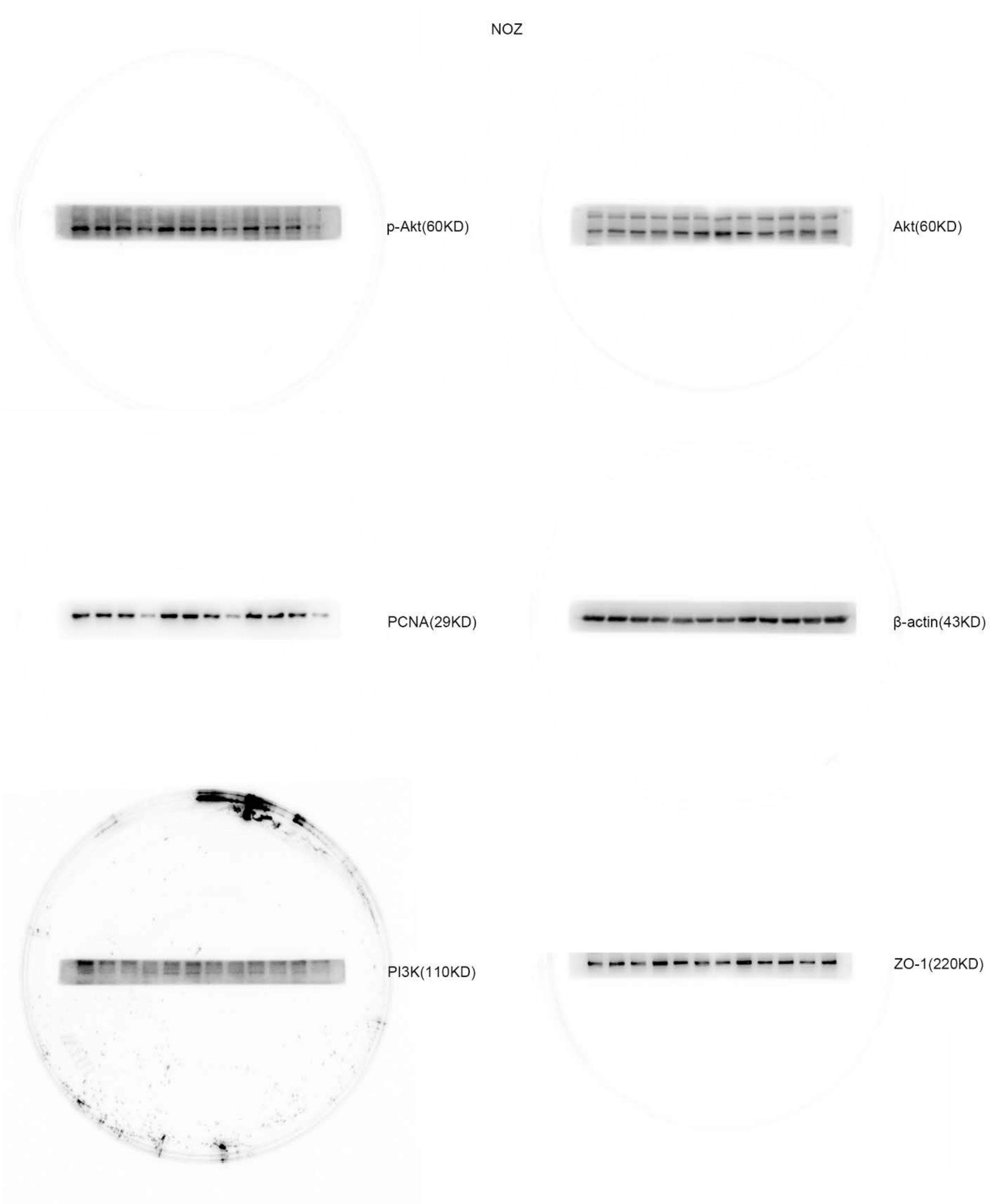
NOZ
p-Akt(60KD)
Akt(60KD)
PCNA(29KD)
β-actin(43KD)
PI3K(110KD)
ZO-1(220KD)

Figure 7

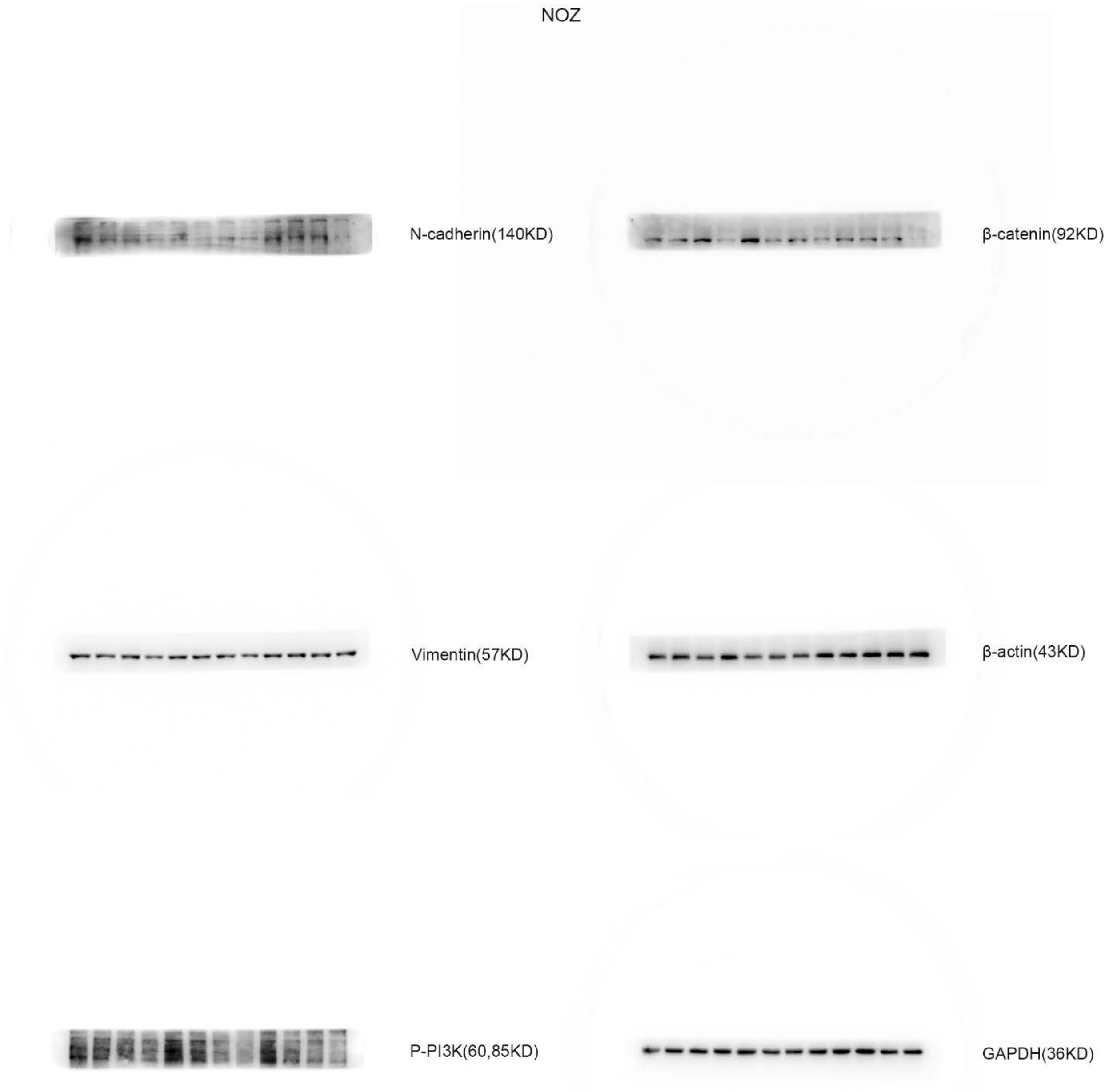
NOZ
N-cadherin(140KD)
β-catenin(92KD)
Vimentin(57KD)
β-actin(43KD)
P-PI3K(60,85KD)
GAPDH(36KD)

Figure 8

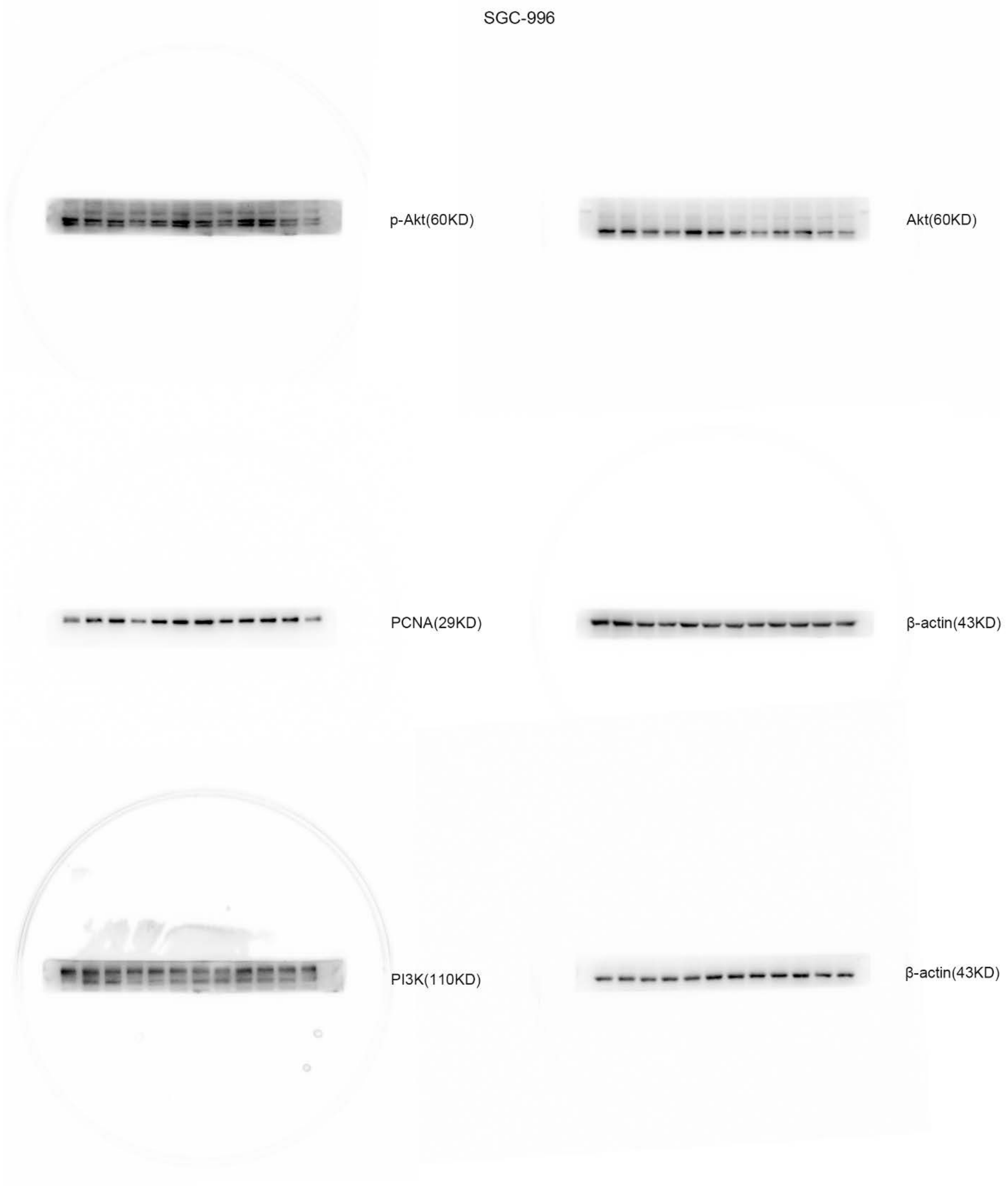
SGC-996
p-Akt(60KD)
Akt(60KD)
PCNA(29KD)
β-actin(43KD)
PI3K(110KD)
β-actin(43KD)

Fig.9

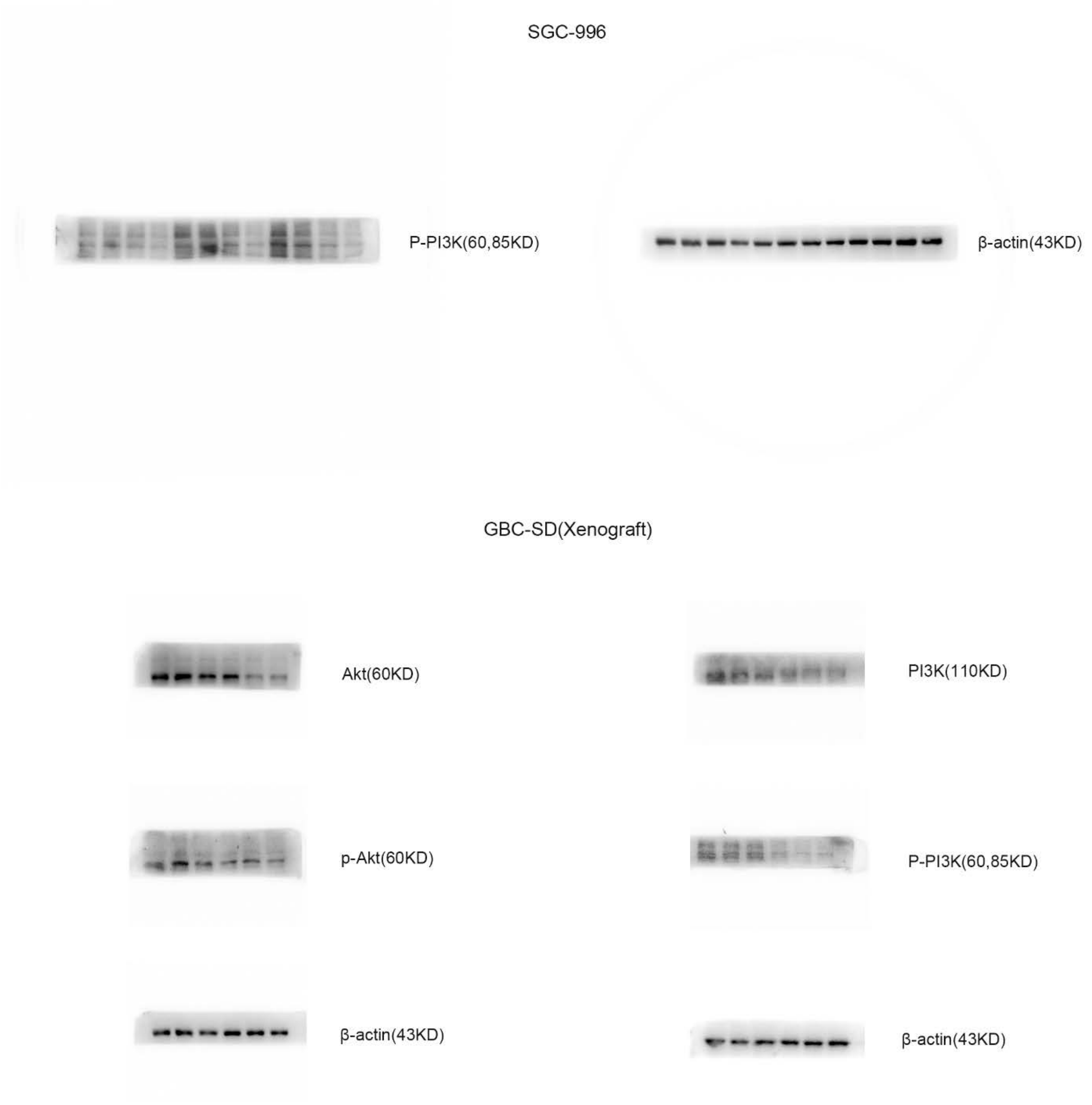

Fig.4-9. The original Western blot figure.
